# Identification of Glaucoma in Diabetics Using the Laguna-ONhE Colourimetric Method and OCT Spectralis

**DOI:** 10.3390/jcm12185876

**Published:** 2023-09-10

**Authors:** Marta Gonzalez-Hernandez, Nisamar Betancor-Caro, Fatima Mesa-Lugo, Ivan Rodriguez-Talavera, Alicia Pareja-Rios, Isabel Guedes-Guedes, Beatriz Estevez-Jorge, Maricela Trujillo-Blanco, Roberto Cordova-Villegas, Juan Espinoza-Gonzalez, Leticia Siguero-Martin, Carolina Goya-Gonzalez, Maria Rodriguez-Dominguez, Daniel Gonzalez-Hernandez, Manuel Gonzalez de la Rosa

**Affiliations:** 1 Instrumentacion y Oftalmologia, INSOFT S.L., 38004 Santa Cruz de Tenerife, Spain; management@insoft.es (D.G.-H.); mgdelarosa1950@gmail.com (M.G.d.l.R.); 2 Hospital Universitario de Canarias, 38320 La Laguna, Spain; nisamarbetancorcaro@gmail.com (N.B.-C.); mesafat@gmail.com (F.M.-L.); ivan.rtalavera@hotmail.com (I.R.-T.); aparejar@gmail.com (A.P.-R.); 3 Hospital Universitario Insular de Gran Canaria, 35016 Las Palmas de Gran Canarias, Spain; isabel.guedes.oft@gmail.com (I.G.-G.); estevez_bea@hotmail.com (B.E.-J.); mtb_oft@yahoo.es (M.T.-B.); juanpabloespinozag@gmail.com (J.E.-G.); leticiasiguero@gmail.com (L.S.-M.); carolgg.93@gmail.com (C.G.-G.); maria281288@hotmail.com (M.R.-D.); 4 Facultad de Medicina, Universidad de Las Palmas de Gran Canaria, 35001 Las Palmas de Gran, Spain

**Keywords:** glaucoma, optic nerve head, diabetes, colourimetry, haemoglobin, perfusion

## Abstract

Background: Previous retrospective results are evaluated prospectively and blinded. Methods: A total of 221 eyes previously classified as normal (G1), 279 as moderate risk of glaucoma (G2) and 217 as high risk (G3) according to the Globin Discriminant Function (GDF) Laguna-ONhE index were examined with OCT Spectralis- Results: In G1, the Bruch’s Membrane Opening Minimum Rim Width (BMO-MRW) was 332 ± 55 microns; in G2, it was 252 ± 47 (*p* < 0.0001); and in G3, 231 ± 44 (*p* < 0.0001). In G1, the 1% and 5% percentiles were 233 and 248, respectively; in G2, they were lower in 28.80% and 42.29% of cases, respectively; and in G3, in 50.23% and 63.59% of cases, respectively. Most of the cases were normal-tension glaucomas. Laguna-ONhE indices showed a curvilinear correlation with BMO-MRW results. The Retinal Nerve Fibre Layer (RNFL) showed a poor relationship with BMO. Assuming G1 to be truly normal, BMO-MRW would have a Receiver operating characteristic (ROC) curve area of 0.901 for G2 and G3 and 0.651 for RNFL. A significant reduction in pixels corresponding to vessels was found in G2 and G3 vs. G1 (*p* < 0.0001). Conclusions: In some cases, these defects appear to be mainly glaucomatous, and in others, they are associated with diabetic microangiopathy. In normal tension glaucoma, RNFL defects may be less severe than those inside the nerve.

## 1. Introduction

In the second half of 2022, our group published two retrospective papers on the optic nerve in diabetes mellitus (DM). In the first one [1], a progressive reduction in the number of pixels corresponding to optic disc vessels was observed over ten years of control in 2153 eyes of 1797 diabetics. This reduction was particularly significant in the superior temporal and inferior temporal sectors, being greater in cases where signs compatible with glaucoma were detected, estimated via colourimetry using our Laguna-ONhE application [2]. Laguna-ONhE uses artificial intelligence and colourimetry techniques to fully automate the identification of normal or glaucoma-like features in retinographies.

In the second of these papers [3], a total of 743,696 images from 313,040 eyes of 173,661 diabetics were retrospectively analysed, reaching a suspicion of glaucoma in 6.70% of them for an intended specificity of 99%. This proportion was higher than the incidence of glaucoma found in the general population when the age of the subject was over 60 years.

A study conducted at the Mayo Clinic describing the characteristics of patients with low-tension glaucoma was published around the same time [4]. Among other factors associated with this type of glaucoma, MD and peripheral vascular disease were identified. Indeed, in the Blue Mountains study [5] and other studies [6], it was suggested that DM is a risk factor for the development of primary open-angle glaucoma (POAG). Subsequently, other studies, which are cited in detail in the Mayo Clinic paper, argued for and against this hypothesis. However, a meta-analysis of 13 papers concluded that DM is indeed a risk factor for glaucoma [7].

It is reasonable to presume that, in the case of diabetics, the existence of specific microangiopathy present in the optic nerve vessels may be involved in some way in the pathophysiology of this specific form of glaucoma. The axons of the ganglion cells, as they pass through the optic nerve head, may be damaged by morphological conditions, such as the characteristics of the lamina cribrosa, and by vascular or axonal flow factors, which may or may not be favoured by the balance between the intraocular pressure and that of the cerebrospinal fluid. Of these causes, ocular pressure may be the main factor in some situations, while in others, as may be the case with diabetics, there may be other more significant influences, especially vascular conditions.

Therefore, some forms of glaucoma in the diabetic patient may present specific features, which may in some ways differentiate them from the much better known and studied POAG.

Our two previous studies were carried out exclusively by analysing the retinographic images accumulated over the years in the Canary Islands Health System for the control of diabetic retinopathy, but we were unaware of other clinical parameters in these patients and, in particular, data that are common in the study of glaucoma. Therefore, it was advisable to corroborate these retrospective results with a prospective study carried out on a significant sample of this population. This would confirm or refute the presumptive diagnoses and, if necessary, establish the type of glaucoma involved.

It was particularly important to verify whether the Laguna-ONhE application was actually detecting, in these patients, defects with characteristics compatible with glaucoma and whether such defects fully or partially corresponded to other morphological parameters that have been more extensively studied in POAG.

The aim of the present study was to confirm the presence of OCT data consistent with the diagnostic suspicion of glaucoma established by Laguna-ONhE and to check whether they showed typical or peculiar characteristics with respect to those common in POAG.

## 2. Materials and Methods

### 2.1. Optic Disc Haemoglobin Measurements

The Laguna-ONhE application analyses photographs of the optic nerve, comparing the colour of the tissue with that of the vessels, to quantify the topographical distribution of haemoglobin [2], and has been fully automated using deep learning methods [8]. It provides one index called Globin Discriminant Function (GDF), which is useful as an identifier of signs of glaucoma disease. Another index, named Globin Individual Pointer (GIP), has better quantitative haemoglobin distribution and perfusion information [9]. For these purposes, a deep learning-based classifier with a high dichotomous component (0 = glaucoma, 1 = normal) is heavily involved in the GDF index, while this same component is less present in the GIP index, which is more oriented towards detecting changes and identifying progression in a particular eye.

Detailed information on the artificial intelligence (AI) methods used to recognise the quality of the images, identify the boundaries of the optic nerve and its vessels, recognise the laterality and estimate the distribution of haemoglobin in the tissue can be found in an appendix to a previously published paper [8].

### 2.2. Subjects

Retisalud is a department of the Public Health Services of the Canary Islands that monitors the possible retinopathy of diabetic patients in its territory by means of periodic retinographies. In a previous study [3], patients without any significant retinopathy were retrospectively analysed. Those requiring strict control (moderate non-proliferative retinopathies) or specific treatment (severe non-proliferative retinopathies, proliferative retinopathies or macular oedema) had previously been excluded from the database and monitored by specific follow-ups.

From the total of 173,661 diabetic patients without clinical retinopathy included in the previous retrospective study, 9000 were randomly selected. One third of them (Group 1, G1) were cases with non-glaucoma results in both eyes and a theoretical specificity of 95% established for a GDF Laguna-ONhE index greater than 0 [2]. Another group (Group 3, G3) were patients with high suspicion of glaucoma in both eyes and a theoretical specificity of 99% (GDF < −15) [1]. Finally, patients in an intermediate situation were included in another group (Group 2, G2), i.e., one eye with non-glaucoma and one eye with probable glaucoma or both eyes with GDF between −15 and 0.

The study protocol adhered to the principles of the 1975 Declaration of Helsinki, revised in 2013, and was approved by the Research Ethics Committee of the Hospital Universitario de Canarias (CHUC_2018_09 (V4)). Patients were contacted consecutively from two hospitals on two different islands, requesting their availability for a glaucoma-oriented examination in a single visit. An attempt was made to balance the number of cases in the three groups. Patients were informed of the characteristics and objectives of this study and signed a written consent form for participation. The patients were examined by specialised healthcare personnel, and the results were tabulated without external intervention and without knowing the characteristics of the group to which each patient belonged. All patients were of Caucasian-Mediterranean descent.

### 2.3. Inclusion and Exclusion Criteria

Inclusion: Visual acuity equal to or greater than 0.5. Spherical equivalent less than 5 dioptres.

Exclusion: Significant diabetic retinopathy or any other pathology affecting vision, except glaucoma. Tonometry by applanation, OCT or retinography impracticable.

### 2.4. Examinations Carried Out

The examination of the patients included anamnesis, visual acuity, anterior pole examination, gonioscopy, Heidelberg OCT (Glaucoma Module Premium-Heidelberg. Mittelgewannweg 27, Heidelberg, Germany), ultrasonic pachymetry, ocular pressure (Goldmann-Haag-Streit AG. Gartenstadtstrasse 10, Köniz, Switzerland), visual field (Humphrey SITA 32-Carl Zeiss Meditec, Inc. 5300 Central Pkwy, Dublin, CA, USA or Octopus 600 TOP 32-Haag-Streit AG. Gartenstadtstrasse 10, Köniz, Switzerland) and colour retinography (Topcon NW400-Topcon. 75-1 Hasunuma-cho, Itabashi-ku, Tokyo, Japan) analysed with the Laguna-ONhE application.

### 2.5. Statistical Analyses

The clinical statistical analyses were performed using the Excel 2016 programme (Excel, Microsoft Corp., Redond, WA, USA) and MedCalc (Version 20.110—64 bits; MedCalc software bvba, Mariakerke, Belgium).

Mean values, standard deviations of absolute and normalised variables, probability values of the differences between groups and correlation between the main variables and ROC (Receiver operating characteristic) analysis were calculated.

## 3. Results

### 3.1. Subjects and Tests Excluded

In total, 93 eyes of 72 patients were excluded for visual acuity lower than 0.5 (45 eyes), impracticable OCTs or retinographies (42 eyes), inconsistent ocular pressure (5 eyes) and high myopia with a spherical equivalent higher than 5D (1 eye).

Most of the patients had no previous perimetric experience and made a large number of catch-trial errors and fixation defects. As there was no possibility to perform further examinations to achieve the desirable “learning effect” due to the unavailability of the volunteers, it was decided to focus this study on morphological and objective parameters.

### 3.2. Description of the Three Groups (Table 1)

Group G1 consisted of 221 eyes (110 left and 111 right) of 151 patients (106 females and 45 males) with a mean age of 65.44 years (SD = 8.54).

G2 consisted of 279 eyes (137 left and 142 right) of 189 patients (118 females and 71 males) with a mean age of 64.83 years (SD = 8.14).

G3 consisted of 217 eyes (104 left and 113 right) of 139 patients (83 females and 56 males) with a mean age of 64.58 years (SD = 8.22).

### 3.3. Results of Each Group

There were no significant differences between age and diabetes duration in the three groups (*p* > 0.1). The IOP was normal in most cases, but previously diagnosed glaucomas were under treatment (Table 1).

**Table 1:** Main results obtained in the three groups. The probability (*p*) of statistical significance of the differences between groups G2 and G3 vs. G1 is indicated.

	AGE	DIABETES (Years)	IOP	PACHYMETRY	GDF	GIP	Vertical C/D Ratio
**G1**	65.4 ± 8.5	11.8 ± 7.4	15.2 ± 3	549.5 ± 36.3	22.6 ± 9.3	48 ± 21.6	0.43 ± 0.11
**G2 (vs. G1)**	64.8 ± 8.1 (*p* = 0.2117)	12.5 ± 8.3 (*p* = 0.1339)	15.5 ± 3.3 (*p* = 0.2086)	538 ± 36.3 (*p* = 0.0002)	−14.5 ± 14.4 (*p* < 0.0001)	−13 ± 26.9 (*p* < 0.0001)	0.61 ± 0.09 (*p* < 0.0001)
**G3 (vs. G1)**	64.5 ± 8.2 (*p* = 0.1419)	11.2 ± 7.8 (*p* = 0.2191)	15.8 ± 3.8 (*p* = 0.0349)	541.7 ± 34.5 (*p* = 0.0106)	−34.3 ± 13.5 (*p* < 0.0001)	−37.4 ± 26.7 (*p* < 0.0001)	0.66 ± 0.09 (*p* < 0.0001)
	**MRW G**	**MRW T**	**MRW ST**	**MRW SN**	**MRW N**	**MRW IN**	**MRW IT**
**G1**	332 ± 55	279 ± 81	336 ± 75	353 ± 81	333 ± 85	388 ± 76	361 ± 75
**G2 (vs. G1)**	252 ± 47 (*p* < 0.0001)	213 ± 64 (*p* < 0.0001)	257 ± 61 (*p* < 0.0001)	262 ± 66 (*p* < 0.0001)	262 ± 73 (*p* < 0.0001)	309 ± 208 (*p* < 0.0001)	274 ± 211 (*p* < 0.0001)
**G3 (vs. G1)**	231 ± 44 (*p* < 0.0001)	193 ± 58 (*p* < 0.0001)	230 ± 52 (*p* < 0.0001)	243 ± 65 (*p* < 0.0001)	240 ± 67 (*p* < 0.0001)	276 ± 64 (*p* < 0.0001)	246 ± 68 (*p* < 0.0001)
	**RNFL G**	**RNFL T**	**RNFL ST**	**RNFL SN**	**RNFL N**	**RNFL IN**	**RNFL IT**
**G1**	100 ± 12	77 ± 18	126 ± 26	117 ± 29	84 ± 21	128 ± 25	135 ± 32
**G2 (vs. G1)**	94 ± 14 (*p* < 0.0001)	81 ± 26 (*p* = 0.0402)	120 ± 28 (*p* = 0.0039)	103 ± 28 (*p* < 0.0001)	89 ± 47 (*p* = 0.0534)	117 ± 33 (*p* < 0.0001)	113 ± 36 (*p* < 0.0001)
**G3 (vs. G1)**	91 ± 14 (*p* < 0.0001)	74 ± 22 (*p* = 0.0487)	115 ± 25 (*p* < 0.0001)	106 ± 27 (*p* < 0.0001)	84 ± 59 (*p* = 0.4929)	116 ± 30 (*p* < 0.0001)	115 ± 35 (*p* < 0.0001)
	**DL glaucoma**	**Hb total**	**Hb cup**	**Cup area**	**Vessels**	**GIP slope (/year)**	**Cup area slope (/year)**
**G1**	0.95 ± 0.06	70.7 ± 3.6	65.0 ± 10.5	25.6% ± 10.0	32.2% ± 3.5	−1.78 ± 4.75	0.57% ± 2.02
**G2 (vs. G1)**	0.55 ± 0.23 (*p* < 0.0001)	65.2 ± 4.1 (*p* < 0.0001)	55.6 ± 8.2 (*p* < 0.0001)	43.3% ± 10.9 (*p* < 0.0001)	30.2% ± 3.9 (*p* < 0.0001)	−3.51 ± 6.13 (*p* = 0.0023)	0.97% ± 2.14 (*p* = 0.043)
**G3 (vs. G1)**	0.32 ± 0.17 (*p* < 0.0001)	64.18 ± 4.27 (*p* < 0.0001)	55.8 ± 7.4 (*p* < 0.0001)	49.4% ± 10.8 (*p* < 0.0001)	30.2% ± 4.1 (*p* < 0.0001)	−4.26 ± 6.92 (*p* = 0.0002)	1.02% ± 3.12 (*p* = 0.068)

G, T, S, N and I = global, temporal, superior, nasal and inferior. DL= deep learning classifier. The slopes were calculated if there were several controls (146 G1, 179 G2 and 155 G3 cases).

Defects in the MRW index were much more evident than those in the fibre layer thickness, where they were limited to the lower and upper sectors.

Other remarkable data are the differences between groups G2 and G3 and group G1 with respect to the deep learning glaucoma classifier, total and cup haemoglobin, cup area, cup/disc vertical ratio and the progressive reduction in the number of pixels corresponding to vessels in the optic disc. It should also be noted that in these groups, there is a greater reduction in the GIP index and an increase in the cup area in successive examinations.

**Figure 1:**
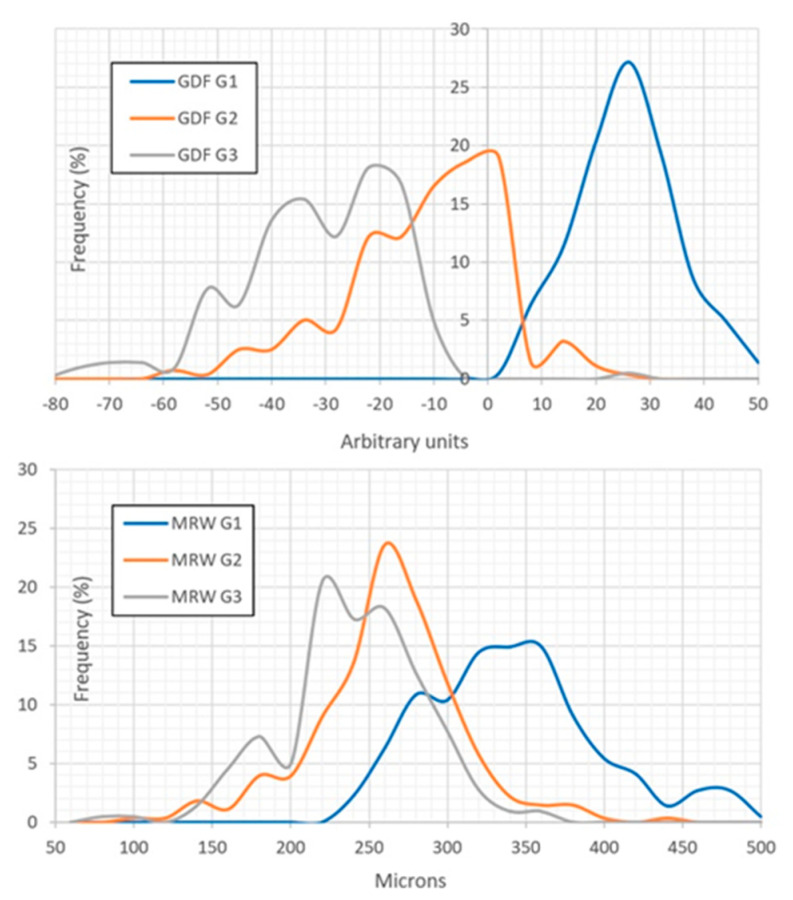
The upper graph shows the frequency distribution of the Laguna-ONhE GDF index in the three groups, and the lower graph shows the distribution of the global BMO-MRW index of the OCT Spectralis.

Figure 1 shows the frequency distribution of the Laguna-ONhE GDF index and the OCT Spectralis global Bruch’s Membrane Opening Minimum Rim Width (BMO-MRW) in the three groups. The mean value of the global BMO-MRW in G1 was 332 microns (SD = 55), while in G2 it was 252 (SD = 47, *p* < 0.0001 relative to G1) and in G3 it was 231 microns (SD = 43, *p* < 0.0001 relative to G1).

In G1, the 1% percentile of the global BMO-MRW was 233 microns, and the 5% percentile was 248 microns. In G2, the BMO-MRW value was below these limits in 28.80% and 42.29% of the cases, respectively. In G3, these percentages were 50.23% and 63.59%, respectively.

In G1, 11 eyes (5.0%) had been previously diagnosed with glaucoma, with a mean global BMO-MRW of 293.1 microns (SD = 46.1), but only 2 of them (0.9%) had a BMO-MRW value of less than 250 microns. Two of the cases had a pachymetry greater than 600 microns associated with BMO-MRW values of 348 and 296 microns. The mean ocular pressure of G1 was 15.3 mmHg (SD = 3.1). Five cases (2.3%) had an IOP higher than 21 mmHg despite being on treatment. None of them had BMO-MRW values lower than 250 microns.

In G2, 37 eyes (13.3%) had been previously diagnosed with glaucoma, with a mean BMO-MRW of 227.5 microns (SD = 53.0), and 27 of them (9.7%) had a BMO-MRW value of less than 250 microns. Two of the cases had a pachymetry greater than 600 microns but were associated with low overall BMO-MRW values of 220 and 248 microns. The mean ocular pressure of G2 was 15.5 mmHg (SD = 3.4, *p* = 0.209 with respect to G1). Additionally, 15 cases (5.4%) had an IOP above 21 mmHg, although 2 of them were on treatment, and 6 had a global BMO-MRW below 250 microns.

In G3, 18 eyes (8.3%) had been previously diagnosed with glaucoma, with a mean global BMO-MRW of 182.1 microns (SD = 61.8), and 14 of them (6.5%) had a BMO-MRW value of less than 250 microns. One case had a pachymetry greater than 600 microns but was associated with BMO-MRW values of 179 microns. The mean ocular pressure was 15.9 mmHG (SD = 3.8, *p* = 0.035 with respect to G1). Additionally, 11 cases (5.1%) had an IOP above 21 mmHg, but only 1 of them was under treatment. All had a global BMO-MRW of less than 250 microns. The 15% percentile global BMO-MRW of G1 matched the 85% percentile of G3 (275 microns).

Assuming the hypothesis that G1 subjects were truly non-glaucomas and G2 and G3 were glaucomas, the global BMO-MRW index would have a Receiver operating characteristic (ROC) curve area of 0.901 and a sensitivity of 51.7% for a specificity of 95%. For the same hypothesis, the Retinal Nerve Fibre Layer (RNFL) would have an ROC area of 0.651 with a sensitivity of 13.4% and a specificity of 95% (*p* < 0.0001 with respect to the global BMO-MRW).

None of the BMO-MRW or RNFL sectors performed significantly better for either of the two global indices.

**Figure 2:**
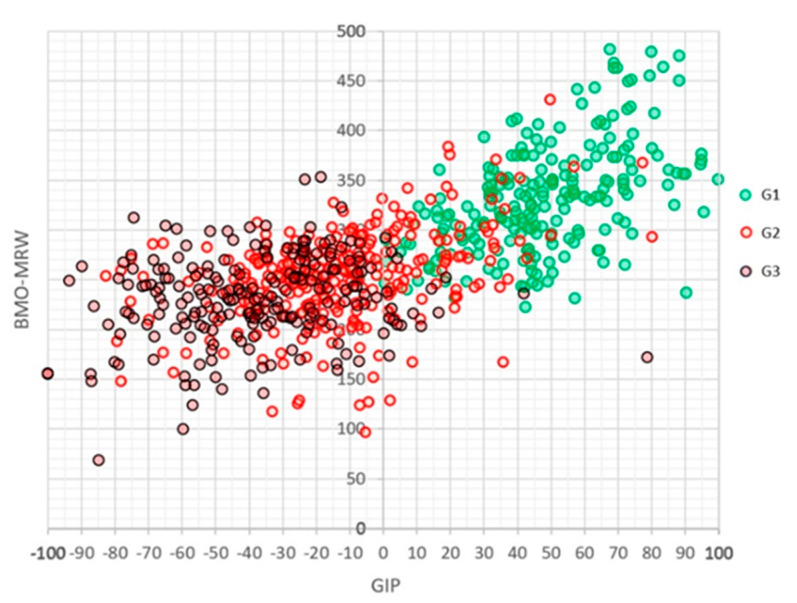
Distribution of Laguna-ONhE GIP values (arbitrary units) and OCT Spectralis global BMO-MRW (microns) in the cases analysed in each group.

Figure 2 shows the relationship between the values of the GIP and global BMO-MRW indices. It can be seen that the G1 cases, represented in green, clearly show higher BMO-MRW thicknesses than the G2 and G3 groups. A second-degree polynomial equation relates both variables with a value of R = 0.707 (*p* < 0.0001): Global BMO-MRW = 262.23 + 1.0043 × GIP + 0.0051 × GIP^2^.

**Figure 3:**
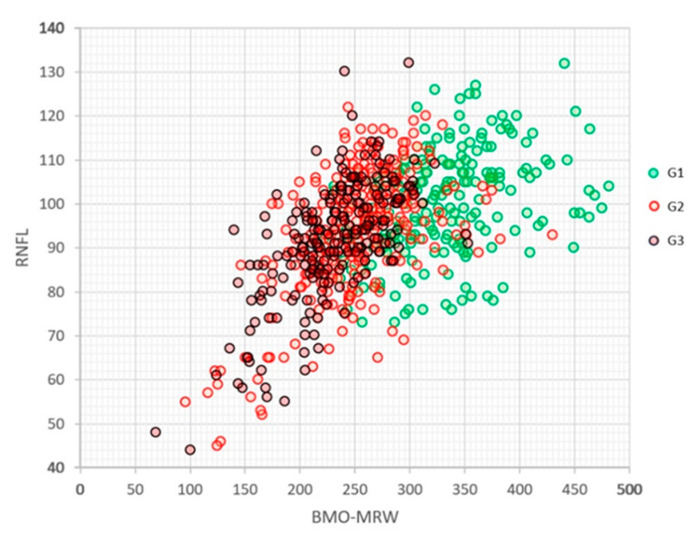
Relationship between global BMO-MRW and global RNFL values of the OCT Spectralis in the three groups analysed.

Figure 3 shows the relationship between the values of the global BMO-MRW and global RNFL indices in the three groups. A significant overlap of the RNFL values was observed, while the BMO-MRW of G1 was better differentiated from those of G2 and G3, although the latter two were intermixed with each other. The linear relationship between both indices was R = 0.556 (*p* < 0.0001), and a good logarithmic relationship would be log(BMO-MRW) = 0.5098 + 0.9677 log(RNFLT) (R = 0.620, *p* < 0.0001).

**Figure 4:**
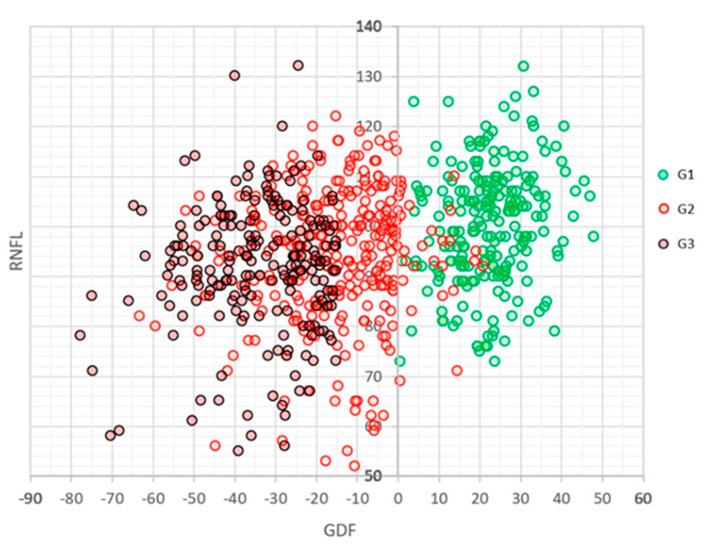
Relationship between Laguna-ONhE GDF values (arbitrary units) and global OCT Spectralis RNFL (microns) in the three groups analysed.

Figure 4 shows the relationship between the values of the GDF and global RNFL indices in the three groups. The relationship is significant but limited (R = 0.087, *p* < 0.0001) for the following linear equation: RNFL = 96.36 + 0.1474 × GDF.

### 3.4. Normalised Data Result

**Figure 5:**
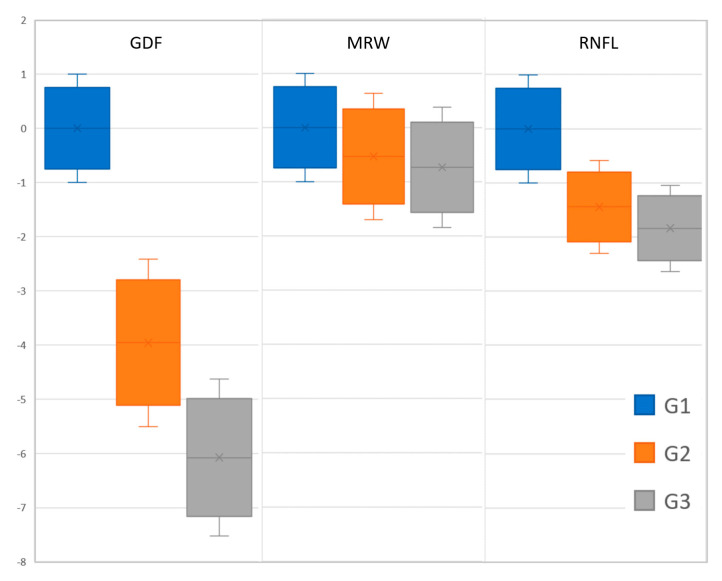
Bar plot comparing the standardised main variables in the three groups.

Normalising the values of the GDF, global BMO-MRW and global RNFL indices (Figure 5) to the mean values and standard deviations obtained in the patients of the G1 series, the mean values and standard deviations of these indices in the three groups were as follows:

G1: 0.00 ± 1.00; 0.00 ± 1.00; 0.00 ± 1.00.

G2: −3.96 ± 1.55; −1.44 ± 0.86; −0.53 ± 1.17.

G3: −6.08 ± 1.44; −1.84 ± 0.80; −0.73 ± 1.11.

**Figure 6:**
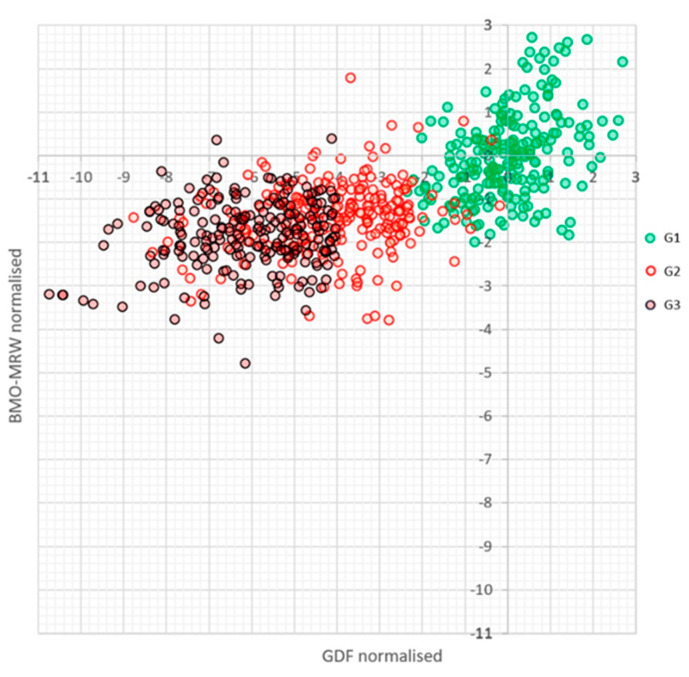
Distribution of Laguna-ONhE GDF and global BMO-MRW values from the OCT Spectralis in the cases analysed in each group, normalised to the mean values and standard deviations of G1.

Figure 6 shows the relationship between the normalised values of the global BMO-MRW and GDF indices in the three groups analysed. It can be seen that, while in G1 the dispersion of both variables is similar, in groups G2 and G3, the range of GDF is much wider than that of BMO-MRW.

## 4. Discussion

BMO-MRW and RNFL values were estimated to decrease with age by 4.0% and 2.1% per decade, respectively [10]. For an age close to 65 years, which is the average age of our patients, a specificity of the global BMO-MRW index of 96.97% was reported for a thickness of 250.08 microns in Caucasian patients [11]. This is highly congruent with the 248 microns at the 5% percentile of the cases that our method selected as non-glaucoma (G1).

There is no agreement in the literature on which OCT Spectralis parameters are better able to identify glaucoma defects. While some authors report the advantages of BMO-MRW over RNFL [12,13,14], others describe the opposite [15] or observe equivalent results [16,17,18].

Some of these differences may be explained by the type of glaucoma being analysed. In the most frequent forms of glaucoma, it has been described that RNFL detects progression better than BMO-MRW [19], but specifically in normal-tension glaucoma, the opposite has been found [20]. In our study, the results were clearly oriented towards the latter conclusion, although it is questionable whether this is a specific feature of all normal-tension glaucomas.

Special care must be taken with the race of the patients, as racial differences can be very large. In normal Korean patients [13] with a mean age of 55.2 years, global BMO-MRW figures as low as 255.2 ± 50.2 microns have been published, and in the Chinese population [16], 288.8 ± 40.7 microns in large discs and 256.9 ± 33.7 in small discs have been found for a similar age. These results are clearly different from those we have observed in the G1 group, which presented thicknesses of 331.8 ± 55.0 microns for a mean age of 65.4 years. In the same direction, a lower usefulness of BMO-MRW has been pointed out in African patients compared with European descendants [21].

On the other hand, although large optical discs have lower BMO-MRW values, this factor does not seem to influence RNFL [22], and for the Laguna-ONhE GDF, this factor is compensated for [3] by using a proprietary calculation of disc size in retinographies [23].

The previous classification of patients was based on the Laguna-ONhE results. Therefore, special care has been taken to avoid biases that may be induced by this. Nevertheless, it can be stated with confidence that the patients in group G1 were truly non-glaucomas. This statement is reasonable, not only because of the frequency of this disease in the general population but also because of the results obtained with OCT in these subjects. Additionally, the few cases in this group that had been previously diagnosed with glaucoma did not show obvious signs of actually suffering from it. It was observed that they corresponded, predictably, to false hypertensions misinterpreted as a consequence of the rigidity of their thick corneas.

A high proportion of patients identified as having glaucoma in the G2 and G3 groups had OCT indices compatible with the disease. These data are congruent with those obtained in other papers in which we compared the Laguna-ONhE results with Cirrus OCT [2,24,25], having recently been corroborated by an independent group in subjects with initial glaucoma [26].

However, in the different studies, there is a notable difference in the results obtained between the Laguna-ONhE and OCT Spectralis RNFL indices. In chronic open-angle glaucoma, an equivalent diagnostic ability between the two indices has been found [27], while another study conducted in Brazil found, as in the present study, that the correlation between the two indices is poor [28]. These data may be related to racial or specific characteristics of normotensive glaucoma, as in the latter, a higher diagnostic ability of BMO-MRW vs. RNFL has been reported [14].

What seems evident, both from the graphical representations of the results and the normalised data, is that the dynamic range of the Laguna-ONhE data is wider than that of OCT Spectralis and that RNFL has, at least in the case of these normal-tension glaucomas, the narrowest of the dynamic ranges. One hypothesis that would explain these results is that the amount of haemoglobin present in the glaucomatous optic nerve would have a smaller floor effect than the morphological OCT data, which are limited by the presence of remaining, non-functioning tissue in the advanced stages of glaucomatous atrophy.

However, it is also possible that the lower alteration in OCT indices is not specifically due to the floor effect but that Laguna-ONhE also detects non-glaucomatous perfusion defects. In previous papers, we pointed out that in diabetic patients, Laguna-ONhE appears to detect defects in nerve microcirculation even in cases without associated signs of glaucoma [1]. Therefore, in some cases, the GDF index may signal perfusion defects without a preferential effect on ganglion cell axons. The MRW index may be more associated with these nerve defects than the nerve fibre layer thickness, as has been found in normal-tension glaucomas [20]. For the same reason, RNFL would be more dissociated than MRW with respect to the Laguna-ONhE-GDF index.

The fact that we did not obtain reliable visual field information in this study can be considered a relative limitation. However, in this type of patient, the visual field provides unreliable information for the study of glaucoma since we now know that diabetic patients may present visual field defects even before clinically evident retinopathy [29].

## 5. Conclusions

A paper was recently published in which five glaucoma experts from five different hospitals and our Laguna-ONhE application interpreted whether 552 fundus images were glaucoma or normal. The Laguna-ONhE’s criteria showed a high degree of agreement with the majority criteria of the experts and were generally higher than the diagnostic match between each of them [30]. The present paper confirms that the Laguna-ONhE criteria are associated with morphological and vascular defects. In glaucoma associated with diabetic vasculopathy, it is likely that the morphological defects of the nerve are relatively dissociated from those of the nerve fibre layer. Future studies with Angio-OCT could complement some aspects of our current research.

## Data Availability

The data presented in this study are available on request from the corresponding author.
